# Epithelioid Malignant Peripheral Nerve Sheath Tumor in the Liver: Report of a Rare Unusual Case with Rhabdoid Morphology and Review of the Literature

**DOI:** 10.1155/2022/8815771

**Published:** 2022-02-18

**Authors:** Benjamin J. Van Treeck, Samar Said, Saba Yasir

**Affiliations:** Department of Laboratory Medicine and Pathology, Division of Anatomic Pathology, Mayo Clinic, Rochester, MN, USA

## Abstract

Malignant peripheral nerve sheath tumor (MPNST) is a rare aggressive type of sarcoma. The epithelioid variant of MPNST has a distinctive morphology and immunophenotype, which can be a diagnostic challenge when it arises in an unusual location. Awareness of these morphologic entities is essential to make an accurate diagnosis. Here, we report a case of epithelioid MPNST involving the liver. The tumor displayed rhabdoid morphology and an unusual immunophenotype. The report also discusses histopathologic features, molecular alterations, and the differential diagnoses of this rare entity.

## 1. Introduction

Epithelioid malignant peripheral nerve sheath tumor (EMPNST) is a rare sarcoma first described in 1954 by McCormack et al. and is considered a variant of malignant peripheral nerve sheath tumor (MPNST) [1]. Both EMPNST and MPNST are derived from peripheral nerve Schwann cells but have differences in morphology, immunophenotype, and molecular biology [2–4]. Treatment for both largely remains the same with a combination of complete resection with negative margins with or without radiation therapy [5]. Chemotherapy may also be considered but is only minimally effective [6].

EMPNST most commonly affects the lower extremity and trunk. Although visceral involvement has been reported, involvement of the liver is extremely rare. The diagnosis of EMPNST largely rests on the morphology and immunohistochemical findings. Epithelioid malignant peripheral nerve sheath tumor can pose a significant diagnostic challenge when it arises in an unusual location, including the liver. Awareness of this rare entity is critical to differentiate from other more common morphologic mimics and to make an accurate diagnosis. To aid surgical pathologists, we present a unique case of EMPNST involving the liver. We will discuss morphologic and immunohistochemical characteristics and the differential diagnoses for this rare entity.

## 2. Case Presentation

### 2.1. Clinical History

A 20-year-old female presented with a 5-month history of shortness of breath and back pain. Laboratory results revealed a normal hemoglobin concentration with microcytosis (hemoglobin 12.4 g/dL, normal 12.1–15.7 g/dL, mean corpuscular volume 80 FL, normal 82-99.6 FL), thrombocytopenia (platelets 117 × 10^3^/UL, normal 140 − 440 × 10^3^/UL), elevated C-reactive protein (113.2 mg/L, normal < 4.9 mg/L), low serum albumin (3.1 g/dL, normal 3.5-5.2 g/dL), elevated alkaline phosphatase (235 U/L, normal 35-104 U/L), normal liver enzymes (AST and ALT), normal lactate dehydrogenase (201 U/L, normal 135-225 U/L), elevated CA125 (69 U/L, normal <34 U/L), and trace proteinuria (30 mg/dL). A CT scan of the thorax and abdomen demonstrated multiple lytic and sclerotic lesions in the T12 vertebral body, L5 vertebral body, sacrum, and right iliac bone ranging from 0.8 to 3.7 cm in greatest dimension (radiology images were not available). The liver appeared to be replaced by innumerable masses ranging up to 11.0 cm in greatest dimension associated with large volume ascites. Few scattered pulmonary nodules measuring up to 0.5 cm were identified in both lungs, associated with right lung atelectasis and pleural effusion. Technetium 99 m SPECT/CT bone scan showed uptake in the previously mentioned lytic bone lesions indicating a high suspicion for bone metastases. Per available clinical information, the patient showed no prior history of any tumors including schwannomas or neurofibromas.

### 2.2. Pathology Review

**Figure 1:**
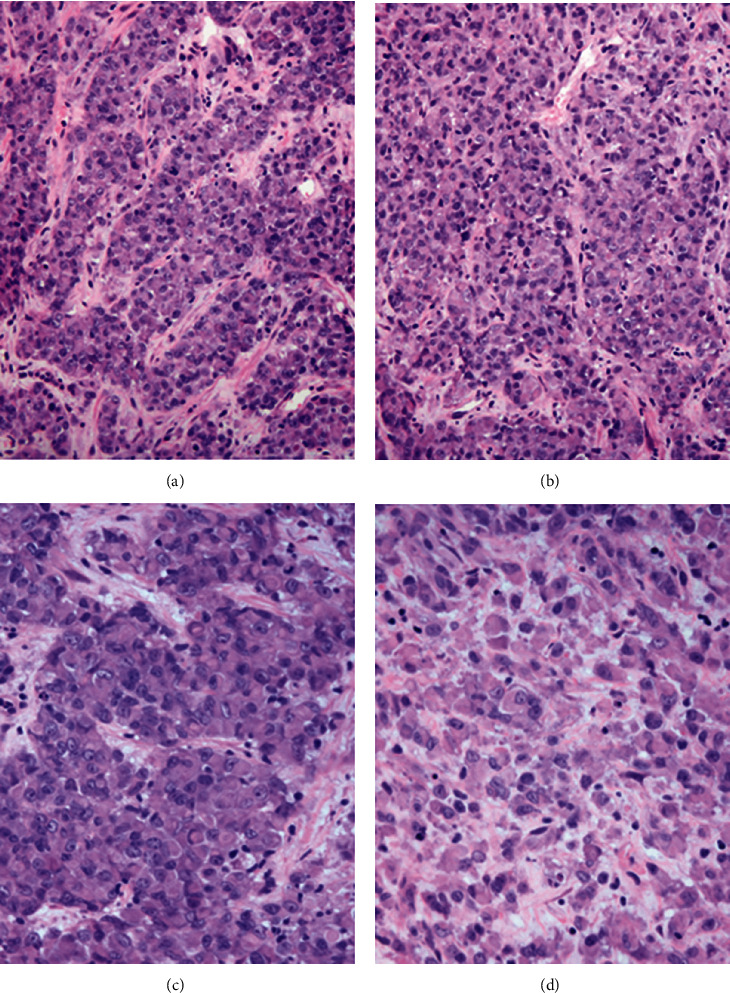
(a, b) Epithelioid malignant peripheral nerve sheath tumor demonstrating nests and cord-like architecture set in a myxoid background (H&E, 200x). (c, d) The individual neoplastic cells exhibit a rhabdoid morphology with eccentric nuclei and conspicuous nucleoli (H&E, 400x).

**Figure 2:**
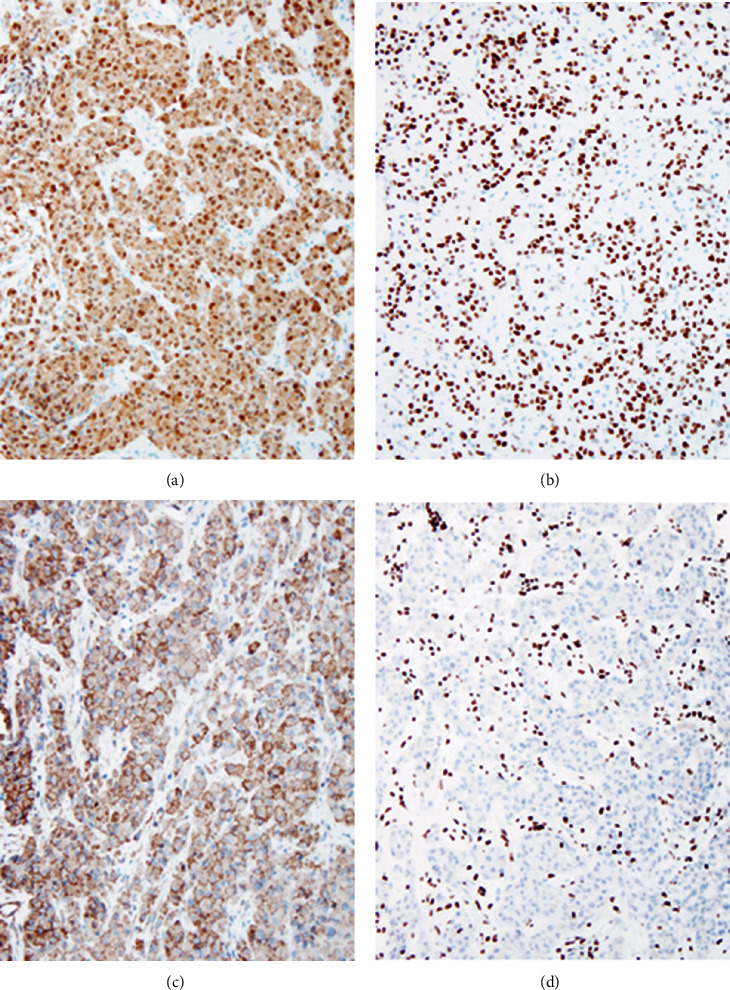
Epithelioid malignant peripheral nerve sheath tumor showing diffuse and strong positivity for S100 (a, 100x), positive staining for SOX10 (b, 100x), weak positivity for smooth muscle actin (c, 100x), and loss of SMARCB1/INI1 with retention in the benign stromal cells (d, 100x).

The single liver needle core biopsy demonstrated a malignant neoplasm purely composed of epithelioid cells. The tumor showed clusters and cords of neoplastic cells located in fibrous stroma (Figures 1(a) and 1(b)). The neoplastic cells were round to polygonal with abundant eosinophilic cytoplasm. The cells had moderate to severe atypia with irregular nuclei with clumped and/or vesicular chromatin and conspicuous nucleoli. Mitoses were readily observed including atypical mitoses. Of note, rhabdoid morphology was appreciated in the majority of tumor cells and characterized by large eccentric vesicular nuclei with prominent nucleoli and abundant amphophilic cytoplasm (Figures 1(c) and 1(d)). No evidence of heterologous/metaplastic elements was present. The neoplastic cells were diffusely and strongly positive for S100 and SOX10 (Figures 2(a) and 2(b)). Smooth muscle actin (Figure 2(c)) and synaptophysin were weakly positive. Immunohistochemical stains for melanocytic differentiation were all negative: melan-A, HMB-45, and tyrosinase. The tumor cells were negative for OSCAR pankeratin, AE1/AE3 pankeratin, chromogranin, trypsin, keratin 7, keratin 20, CDX2, estrogen receptor, PAX8, GATA3, myogenin, MYOD1, CD31, CD34, ERG, desmin, c-KIT, and DOG-1. SMARCB1*/*INI1 expression was lost in the neoplastic cells but retained in the stromal cells, indicating a good positive internal control (Figure 2(d)). SMARCA4/BRG1 was retained. Fluorescent in situ hybridization with break apart probes for the EWSR1 gene region was also performed and was negative for EWSR1 gene rearrangements.

### 2.3. Clinical Follow-Up

Unfortunately, the patient was lost to follow-up, and no information is available regarding treatment or outcome.

## 3. Discussion

EMPNST is a rare sarcoma and is considered a variant of MPNST with histopathologic differences as listed in Table 1 [2–4, 7]. EMPNST has an equal sex distribution and presents most commonly in the third or fourth decade of life with a broad age range from 6 to 80 years old. The most common site of origin is the lower extremity followed by the upper extremity, trunk, and subfascial locations such as the ileum, prostate, pleura, mediastinum, and retroperitoneum [2, 8, 9]. The majority of cases occur in the subcutaneous tissue rather than deep or visceral locations. EMPNST can undergo recurrence at the site of excision and distant metastasis with the reported locations including the lung, bone, brain, lymph node, and adrenal gland. The liver is a common site for involvement by mesenchymal neoplasms/sarcomas, including angiosarcomas, epithelioid hemangioendothelioma, and gastrointestinal stromal tumor. However, liver involvement by EMPNST is an extremely rare finding. A ten-year search of the pathology database at Mayo Clinic in Rochester, Minnesota, showed no additional internal cases of EMPNST involving the liver. Due to the rarity of this neoplasm in the liver and significant morphologic and immunohistochemical overlap with other entities, accurate diagnosis can be challenging. Therefore, awareness of key morphologic and immunohistochemical features is critical to avoid misdiagnosis.

EMPNST is characterized by a predominantly epithelioid cytomorphology and frequent multilobulated growth pattern. The tumor displays various morphologic characteristics, including circumscribed solid sheet-like growth pattern, prominent fibromyxoid stroma, prominent multinucleated giant cells, clear cell morphology, nuclear inclusions, perivascular whorling, and heterologous/metaplastic elements such as cartilage or bone [10]. Rhabdoid cytomorphology, as seen in our case, has also been reported in a minority of cases [10].

The molecular biology of EMPNST has been recently elucidated by Schaefer et al. demonstrating tumor suppressor gene *SMARCB1/INI1* inactivating mutations present in the majority (75%) of EMPNST correlating with SMARCB1/INI1 immunohistochemical loss [10]. Loss of SMARCB1/INI1 leads to aberrant SWItch sucrose non-fermentable (SWI/SNF) complexes preventing proper chromatin remodeling. Additionally, uninhibited EZH2 methyltransferase overactivity results from loss of antagonism by SMARCB1/INI1. Studies utilizing EZH2 inhibitors in patients with epithelioid sarcoma, which is also SMARCB1/INI1 deficient, and SMARCB1/INI1-deficient rhabdoid tumor have shown improved survival [11]. Therefore, EZH2 inhibitors may be beneficial in EMPNST but will require testing to ascertain benefit in EMPNST patients. No other targetable therapies are currently known to be of benefit in patients with EMPNST.

Of the cases without *SMARCB1/INI1* inactivating mutations, mutations in *NF1*, *NF2*, *CDKN2A*, and *TP53* were identified. Chromosomal copy number variations were also identified by next-generation sequencing with losses on chromosome 22q, which is the location of the *SMARCB1/INI1* gene. Chromosome 22q deletions are the most common chromosomal aberration present in 50% of EMPNST cases. EMPNST lacks inactivating mutations in *SUZ12* and *EED*, translating into retained H3K27me3 expression contrasting with H3K27me3 loss in 50% of MPNST [10, 12]. Important to note from the molecular analysis and clinicopathologic characteristics is the infrequent association of EMPNST with neurofibromatosis type 1, in contrast to MPNST [2, 8, 9].

The differential diagnoses for EMPNST include several entities that have morphologic and immunophenotypic similarities (Table 2). Malignant melanoma can morphologically mimic any tumor, but usually will have more cytologic atypia compared to EMPNST, so it is always important to consider melanoma and rule it out with appropriate immunostains including melan-A, HMB-45, and MiTF. It is important to remember that both EMPNST and melanoma will be positive for S100 and SOX10. However, EMPNST will be negative for the more specific melanocytic markers such as melan-A, HMB-45, and MiTF, while most malignant melanomas are positive. Another important immunostain to utilize in the differentiation of EMPNST from melanoma is SMARCB1/INI1 which is retained in melanoma but absent in 50-70% of EMPNST [2, 13].

Myoepithelial carcinoma can present morphologically similar to EMPNST with both having nests/cords of epithelioid neoplastic cells in a background of myxoid and/or fibrous matrix. Rhabdoid morphology has even been reported in myoepithelial carcinoma further complicating the histologic overlap [14]. Furthermore, these two entities share a similar immunophenotype with expression of S100 and absence of SMARCB1/INI1. However, 50% of myoepithelial carcinomas possess *EWSR1* rearrangements with *PBX1*, *ZNF444*, or *POU5F1* which are not seen in EMPNST, making cytogenetics very useful to differentiate these two entities [15–17]. In addition, myoepithelial carcinoma expresses cytokeratins and p63 (a myoepithelial marker) more commonly than in EMPNST which may also help to differentiate these two entities, especially in myoepithelial carcinoma cases without *EWSR1* rearrangement.

Malignant rhabdoid tumor, also known as atypical teratoid/rhabdoid tumor of the central nervous system if occurring in the central nervous system, is included in the differential due to its hallmark rhabdoid morphology and absence of SMARCB1/INI1 expression [18]. Malignant rhabdoid tumor typically shows patchy S100 positivity, whereas EMPNST possesses strong and diffusely positive S100 expression [2, 8, 18]. Age and cytokeratin expression may also be helpful to differentiate malignant rhabdoid tumor from EMPNST as the former usually occurs in children less than or equal to 3 years of age and marks with cytokeratin while EMPNST typically present in the third or fourth decade and are cytokeratin negative [7, 8, 19]. Cytogenetics and mutational analysis may not be helpful to differentiate these two entities as both have inactivating mutations of *SMARCB1/INI1* and chromosomal aberrations of 22q [10, 20–22].

Malignant gastrointestinal neuroectodermal tumor should also be entertained in the differential diagnosis of EMPNST. On histology, malignant gastrointestinal neuroectodermal tumor may demonstrate osteoclast-like giant cells which may be a clue to the diagnosis, but some cases may not possess osteoclast-like giant cells. Immunohistochemically, SMARCB1/INI1 is retained in malignant gastrointestinal neuroectodermal tumor which is a helpful feature, and cytogenetically, this entity displays *EWSR1* rearrangements with *CREB1*, *ATF1*, and other unknown gene partners [23, 24].

Finally, epithelioid sarcoma can histologically mimic EMPNST, and it can even display rhabdoid morphology [25]. SMARCB1/INI1 loss is also frequent (approximately 90%) in both conventional and proximal types of epithelioid sarcoma due to deletions of the *SMARCB1/INI1* gene on chromosome 22q, making the distinction between these two entities difficult [13, 26]. However, immunohistochemistry can help differentiate these two entities based on S100 and SOX10 negativity and cytokeratin positivity in epithelioid sarcoma in contrast to EMPNST.

## 4. Conclusion

Here, we presented a unique case of EMPNST with rhabdoid morphology in the liver. Ascertaining the diagnosis of this tumor can be challenging given the significant morphologic and immunophenotypic overlap with other malignancies. However, with the aid of immunohistochemistry and cytogenetics, the correct diagnosis can be elucidated.

## Figures and Tables

**Table 1:** Histopathologic comparison of MPNST and EMPNST.

	MPNST	EMPNST
Morphology:	Spindle cell predominate	Epithelioid cell predominant
Immunohistochemistry:		
SOX10	Patchy positive	Diffusely positive
S100	Negative to scattered positivity	Diffusely positive
H3K27me3	Lost in 50%	Retained
SMARCB1/INI1	Retained	Lost in 50-70%
Inactivating mutations:		
*SMARCB1/INI1*	Absent	Present in 75%
*NF1*	Frequent	Occasionally present
*NF2*	Rare	Occasionally present
*CDKN2A*	Frequent	Occasionally present
*TP53*	Frequent	Occasionally present
*SUZ12 and EED*	Frequent	Absent

Abbreviations: MPNST: malignant peripheral nerve sheath tumor; EMPNST: epithelioid malignant peripheral nerve sheath tumor.

**Table 2:** Immunophenotype of neoplasms in the differential diagnosis of epithelioid malignant peripheral nerve sheath tumor.

	Neoplasm					
	EMPNST	Malignant rhabdoid tumor	Myoepithelial carcinoma	Epithelioid sarcoma	Malignant melanoma	MGNET
*Immunostain*						
INI1	Absent (50-70%)	Absent (98%)	Absent (10-40%)	Absent (90%)	Retained	Retained
Keratin	Rare	Positive	Positive	Positive	Negative	Negative
EMA	Rare	Positive	Positive	Positive	Negative	Negative
SMA	Negative∗	Variable	Variable	Negative	Negative	Negative
Desmin	Negative	Variable	Negative	Negative	Negative	Negative
Myogenin	Negative	Negative	Negative	Negative	Negative	Negative
MelanA	Negative	Negative	Negative	Negative	Positive	Negative
HMB45	Negative	Negative	Negative	Negative	Positive	Negative
SOX10	Positive	Negative	Positive	Negative	Positive	Positive
S100	Positive	Variable	Positive	Negative	Positive	Positive
GFAP	Negative	Variable	Negative	Negative	Negative	Negative
CD34	Negative	Negative	Negative	Variable	Negative	Negative

^∗^EMPNST with rhabdoid differentiation may express SMA. ^Please note that the immunophenotypes for the neoplasms listed above may deviate from what is listed. Abbreviations: EMPNST: epithelioid malignant peripheral nerve sheath tumor; MGNET: malignant gastrointestinal neuroectodermal tumor; EMA: epithelial membrane antigen; SMA: smooth muscle actin; GFAP: glial fibrillary acidic protein.

## Abbreviations

EMPNST: Epithelioid malignant peripheral nerve sheath tumor
MPNST: Malignant peripheral nerve sheath tumor
cm: Centimeters.

## References

[1] Mc C. L., Hazard J. B., Dickson J. A. (1954). Malignant epithelioid neurilemoma (schwannoma). *Cancer*.

[2] Jo V. Y., Fletcher C. D. M. (2017). SMARCB1/INI1 loss in epithelioid schwannoma: a clinicopathologic and immunohistochemical study of 65 cases. *The American Journal of Surgical Pathology*.

[3] Lee W., Teckie S., Wiesner T. (2014). PRC2 is recurrently inactivated through _EED_ or _SUZ12_ loss in malignant peripheral nerve sheath tumors. *Nature Genetics*.

[4] Birindelli S., Perrone F., Oggionni M. (2001). Rb and TP53 pathway alterations in sporadic and NF1-related malignant peripheral nerve sheath tumors. *Laboratory Investigation*.

[5] Dunn G. P., Spiliopoulos K., Plotkin S. R. (2013). Role of resection of malignant peripheral nerve sheath tumors in patients with neurofibromatosis type 1. *Journal of Neurosurgery*.

[6] Pervaiz N., Colterjohn N., Farrokhyar F., Tozer R., Figueredo A., Ghert M. (2008). A systematic meta-analysis of randomized controlled trials of adjuvant chemotherapy for localized resectable soft-tissue sarcoma. *Cancer*.

[7] Jo V. Y., Fletcher C. D. (2015). Epithelioid malignant peripheral nerve sheath tumor: clinicopathologic analysis of 63 cases. *The American Journal of Surgical Pathology*.

[8] Laskin W. B., Weiss S. W., Bratthauer G. L. (1991). Epithelioid variant of malignant peripheral nerve sheath tumor (malignant epithelioid schwannoma). *The American Journal of Surgical Pathology*.

[9] Du P., Zhu J., Zhang Z. D. (2019). Recurrent epithelioid malignant peripheral nerve sheath tumor with neurofibromatosis type 1: a case report and literature review. *Oncology Letters*.

[10] Schaefer I. M., Dong F., Garcia E. P., Fletcher C. D. M., Jo V. Y. (2019). Recurrent SMARCB1 inactivation in epithelioid malignant peripheral nerve sheath tumors. *The American Journal of Surgical Pathology*.

[11] Schaefer I. M., Hornick J. L. (2021). SWI/SNF complex-deficient soft tissue neoplasms: an update. *Seminars in Diagnostic Pathology*.

[12] Schaefer I. M., Fletcher C. D., Hornick J. L. (2016). Loss of H3K27 trimethylation distinguishes malignant peripheral nerve sheath tumors from histologic mimics. *Modern Pathology*.

[13] Hornick J. L., Dal Cin P., Fletcher C. D. (2009). Loss of INI1 expression is characteristic of both conventional and proximal-type epithelioid sarcoma. *The American Journal of Surgical Pathology*.

[14] Thway K., Bown N., Miah A., Turner R., Fisher C. (2015). Rhabdoid variant of myoepithelial carcinoma, with EWSR1 rearrangement: expanding the spectrum of EWSR1-rearranged myoepithelial tumors. *Head and Neck Pathology*.

[15] Antonescu C. R., Zhang L., Chang N. E. (2010). EWSR1-POU5F1 fusion in soft tissue myoepithelial tumors: a molecular analysis of sixty-six cases, including soft tissue, bone, and visceral lesions, showing common involvement of the EWSR1 gene. *Genes, Chromosomes & Cancer*.

[16] Agaram N. P., Chen H. W., Zhang L. (2015). EWSR1-PBX3: a novel gene fusion in myoepithelial tumors. *Genes, Chromosomes & Cancer*.

[17] Huang S. C., Chen H. W., Zhang L. (2015). Novel FUS-KLF17 and EWSR1-KLF17 fusions in myoepithelial tumors. *Genes, Chromosomes & Cancer*.

[18] Burger P. C., Yu I. T., Tihan T. (1998). Atypical teratoid/rhabdoid tumor of the central nervous system: a highly malignant tumor of infancy and childhood frequently mistaken for medulloblastoma: a Pediatric Oncology Group study. *The American Journal of Surgical Pathology*.

[19] Agaimy A. (2020). What is new in epithelioid soft tissue tumors?. *Virchows Archiv*.

[20] Biegel J. A., Tan L., Zhang F., Wainwright L., Russo P., Rorke L. B. (2002). Alterations of the hSNF5/INI1 gene in central nervous system atypical teratoid/rhabdoid tumors and renal and extrarenal rhabdoid tumors. *Clinical Cancer Research*.

[21] Douglass E. C., Valentine M., Rowe S. T. (1990). Malignant rhabdoid tumor: a highly malignant childhood tumor with minimal karyotypic changes. *Genes, Chromosomes & Cancer*.

[22] Schofield D. E., Beckwith J. B., Sklar J. (1996). Loss of heterozygosity at chromosome regions 22q11-12 and 11p15.5 in renal rhabdoid tumors. *Genes, Chromosomes & Cancer*.

[23] Stockman D. L., Miettinen M., Suster S. (2012). Malignant gastrointestinal neuroectodermal tumor: clinicopathologic, immunohistochemical, ultrastructural, and molecular analysis of 16 cases with a reappraisal of clear cell sarcoma-like tumors of the gastrointestinal tract. *The American Journal of Surgical Pathology*.

[24] Green C., Spagnolo D. V., Robbins P. D., Fermoyle S., Wong D. D. (2018). Clear cell sarcoma of the gastrointestinal tract and malignant gastrointestinal neuroectodermal tumour: distinct or related entities? A review. *Pathology*.

[25] Chbani L., Guillou L., Terrier P. (2009). Epithelioid sarcoma. *American Journal of Clinical Pathology*.

[26] Sullivan L. M., Folpe A. L., Pawel B. R., Judkins A. R., Biegel J. A. (2013). Epithelioid sarcoma is associated with a high percentage of _SMARCB1_ deletions. *Modern Pathology*.

